# Nutrition Recommendations for Bodybuilders in the Off-Season: A Narrative Review

**DOI:** 10.3390/sports7070154

**Published:** 2019-06-26

**Authors:** Juma Iraki, Peter Fitschen, Sergio Espinar, Eric Helms

**Affiliations:** 1 Iraki Nutrition AS, 2008 Fjerdingby, Norway; 2 Fitbody and Physique LLC, Stevens Point, WI 54481, USA; 3 Sport Performance Research Institute New Zealand (SPRINZ) at AUT Millennium, Auckland University of Technology, Auckland 0632, New Zealand

**Keywords:** Bodybuilding, nutrition, muscle hypertrophy

## Abstract

Many nutrition practices often used by bodybuilders lack scientific support and can be detrimental to health. Recommendations during the dieting phase are provided in the scientific literature, but little attention has been devoted to bodybuilders during the off-season phase. During the off-season phase, the goal is to increase muscle mass without adding unnecessary body fat. This review evaluated the scientific literature and provides nutrition and dietary supplement recommendations for natural bodybuilders during the off-season phase. A hyper-energetic diet (~10–20%) should be consumed with a target weight gain of ~0.25–0.5% of bodyweight/week for novice/intermediate bodybuilders. Advanced bodybuilders should be more conservative with the caloric surplus and weekly weight gain. Sufficient protein (1.6–2.2 g/kg/day) should be consumed with optimal amounts 0.40–0.55 g/kg per meal and distributed evenly throughout the day (3–6 meals) including within 1–2 hours pre- and post-training. Fat should be consumed in moderate amounts (0.5–1.5 g/kg/day). Remaining calories should come from carbohydrates with focus on consuming sufficient amounts (≥3–5 g/kg/day) to support energy demands from resistance exercise. Creatine monohydrate (3–5 g/day), caffeine (5–6 mg/kg), beta-alanine (3–5 g/day) and citrulline malate (8 g/day) might yield ergogenic effects that can be beneficial for bodybuilders.

## 1. Introduction

Bodybuilding is more than a sport; it is an art and culture. It differentiates itself from performance sports as the athletes are judged on appearance rather than athletic ability on competition day. Bodybuilders pose onstage where they are judged on muscularity, definition, and symmetry. During a season, bodybuilders go through three different phases: muscle-gaining phase (off-season), dieting for competition (contest preparation) and the competition itself. Most of the literature surrounds the dieting phase [1]. 

However, the scientific literature on dietary recommendations for bodybuilders in the off-season is lacking. This is an important gap, as most of a bodybuilder’s career is spent in this phase where the goal is to increase muscle mass while minimizing excess increases in fat mass. Bodybuilders are known for having rigid attitudes toward food selection, meal frequency, nutrition timing and supplementation [2]. Historically, information about nutrition and supplementation has been passed on by bodybuilding magazines and successful competitors, but recently more information has emerged via the internet and forums [3,4]. As such, many of the dietary strategies used by bodybuilders do not have sound scientific support and there is evidence in the scientific literature that a number of these strategies, including the heavy use of dietary supplements, can be detrimental to health [5,6,7]. 

Since bodybuilders spend most of their time in the off-season, there is a clear need for safe and evidence-based nutrition and dietary supplement recommendations for this population. There is also evidence that some bodybuilders, especially high-level competitors in natural bodybuilding, may be interested in evidence-based information [8]. The purpose of this review is to evaluate the scientific literature on topics related to nutrition and dietary supplementation relevant for bodybuilders in the off-season and provide practical recommendations for energy intake, macronutrients, meal frequency, nutrient timing and dietary supplements.

## 2. Energy

During the off-season, the main goal of a bodybuilder is to increase muscle mass while minimizing increases in fat mass through the use of resistance training and maintaining a positive energy balance. In order to accurately assess energy requirements for bodybuilders during the off-season, training volume, frequency and intensity must be considered. During the off-season phase, it has been reported that bodybuilders resistance train 5–6 times a week, exercising each muscle group 1–2 times weekly [9]. It was also reported that they follow a high-volume training routine with 4–5 exercises per muscle group, performing 3–6 sets per exercise, 7–12 repetition maximum (RM) for each set with 1–2 min rest between sets. Training session duration was reported as ~40–90 min. However, training plans can differ greatly from athlete to athlete. The average calorie intake of bodybuilders must also be evaluated. In the off-season, energy intake is usually substantially higher compared to the dieting phase with dietary intakes among male bodybuilders being reported at an average intake of ~3800 kcal/day during the off-season and ~2400 kcal/day during the dieting phase [2]. Due to the limited information available on nutritional strategies during the off-season phase, this review will discuss optimizing strategies during this phase. However, readers are encouraged to read the review by Helms and colleagues on the dieting phase which also covers recommendations for macronutrients, meal frequency and nutrient timing as well as dietary supplements [1].

### Positive Energy Balance

Positive energy balance has been shown to have an important anabolic effect, even in the absence of resistance training [10]. However, combining a positive energy balance with resistance training provides the most effective method to ensure the anabolic effects are directed toward increasing skeletal muscle mass [11,12]. The ideal size of the energy surplus to gain lean mass while limiting the accumulation of adipose tissue may differ based upon training status. In untrained subjects, a substantial energy surplus of ~2000 kcal combined with resistance training has been shown to provide robust weight gain where the contribution from lean body mass (LBM) can be as high as 100% [12]. However, in trained subjects, substantial energy surpluses might not be necessary or beneficial. One study conducted on elite athletes looked at the effect of dietary guidance on body composition changes among elite athletes when resistance training was combined with different energy surplus magnitudes. One group with an average bodyweight of 75 kg, consumed energy ad libitum (2964 kcal) to reach a very small surplus, while a second group with an average body weight of 71 kg received dietary counseling and consumed ~600 kcal more than the ad libitum group [13].

Both groups followed the same 4-days per week resistance training program over a period of 8–12 weeks. The researchers hypothesized that the hyper-energetic group would have greater gains in body weight and LBM. Although the hyper-energetic group achieved greater increases in LBM compared to those eating ad libitum, this failed to reach statistical significance (1.7 kg vs. 1.2 kg, respectively). Further, compared to the ad libitum group they had significantly larger increases in fat mass (1.1 kg vs. 0.2 kg, respectively). The researchers concluded that a 200–300 kcal per day surplus in highly trained athletes might be more appropriate than 500 kcal to minimize the risk of unnecessary increases in body fat. Untrained subjects, further from their genetic ceiling of muscle mass, may be able to gain muscle at a faster rate compared to trained individuals. 

Rates of muscle growth may slow as an individual becomes more advanced [14]. Thus, larger energy surpluses may be more beneficial for novice bodybuilders, while advanced bodybuilders might benefit more from conservative hyper-energetic diets to limit unnecessary increases in body fat. Previous studies have recommended bodybuilders to consume a slightly hyper-energetic diet with a ~15% increase in energy intake above maintenance in the off-season [15]. However, this does not take into consideration the training history and experience level of the individual bodybuilder. Because the ability to gain muscle mass is limited, an aggressive surplus can result in an unnecessary gain of body fat, which would increase the duration or the severity of subsequent contest prep periods, consequentially increasing the duration or severity of low energy availability. Thus, the number of calories a bodybuilder consumes above maintenance may need to be set based on experience level, then adjusted based on rate of weight gain and changes in body composition. Given that bodybuilders often experience rapid weight gain after a competition, it might be beneficial to have a target for weight gain per week and adjust accordingly [16,17]. 

However, initially post competition, a faster weight gain to help restore a competitor to a healthy status both psychologically and physiologically might be beneficial before the rate of weight gain is slowed to limit excessive accumulation of adipose tissue. In the scientific literature, it has been recommended to aim for a target weight gain of ~0.25–0.5 kg per week when trying to increase LBM and minimize gains in fat mass [14,18]. For the advanced bodybuilder, a potential 2 kg increase in body weight on a monthly basis might be too excessive and result in unnecessary accrual of body fat; thus, this rate should be considered with caution. Based on the current evidence, it may be appropriate to recommend bodybuilders to consume a slightly hyper-energetic diet (~10–20% above maintenance calories) in the off-season and recommend advanced bodybuilders to aim for the lower end of this recommendation, or even be more conservative if substantial increases in fat mass are experienced. Given that bodybuilders on average consume 45 kcal/kg during the off-season, the recommended surplus would equate to approximately 42–48 kcal/kg [2]. Aiming for a target weight gain of ~0.25–0.5% of bodyweight per week might be useful, while also adjusting energy intake based on changes in body composition. In addition, it may be more appropriate to look at average weekly weight changes based on daily (or multiple times per week) weigh ins to limit the errors of daily fluctuations of weight that may occur during the week. Once caloric surplus is determined, the next step would be to distribute the calories between protein, fats and carbohydrates.

## 3. Protein 

Skeletal muscle protein turnover is the relationship between muscle protein synthesis (MPS) and muscle protein breakdown (MPB). Skeletal muscle hypertrophy requires a net balance where MPS exceeds MPB. Resistance exercise provides the initiating tension stimulus that drives hypertrophy resulting from cumulative increases in MPS after chronic resistance exercise [19]; however, increases in fat free mass (FFM) can be limited if an insufficient daily protein intake is consumed [20]. In addition to the total amount consumed per day, researchers have speculated that the quality of protein may augment resistance training-induced muscle gain [21]. Thus, both of these topics will be discussed in the following sections. 

### 3.1. Daily Intake

While the current RDA for protein in healthy individuals is 0.8 g/kg, twice this amount was observed to maximize resistance training-induced hypertrophy in a 2018 meta-analysis by Morton and colleagues [22]. Furthermore, the authors noted “it may be prudent to recommend ~2.2 g protein/kg/d for those seeking to maximize resistance training-induced gains in FFM”, as 2.2 g/kg was the upper end of the confidence limit [22] and individual differences dictate that some athletes will have higher protein needs than others [23]. Additionally, a “better safe than sorry” recommendation is likely safe given the lack of apparent harm over 1–2 year trials among lifters consuming protein intakes of at least 2.2 g/kg [24,25]. Finally, the mean and upper 95% confidence limit for protein requirements using the indicator amino acid oxidation technique among male bodybuilders on non-training days, were reported as 1.7 and 2.2 g/kg [26], respectively—which is similar to the requirement among women when normalized to FFM [27]. 

However, bodybuilders have been reported to consume up to 4.3 g/kg of protein per day among males, and 2.8 g/kg among females which far exceeds these recommendations [2]. Guidelines previously given for bodybuilders in the off-season, were to consume 25–30% of their energy intake from protein [15]. It might be reasonable to argue against giving recommendations based on percentages of total energy intake, due to the fact that a light individual with high energy requirements might end up consuming protein which far exceeds what is necessary and required. Further, this can also lead to insufficient intakes of carbohydrates and fats if an athlete is targeting a specific caloric intake. Thus, recommending protein requirements based on body weight might be more appropriate. Therefore, bodybuilders should consume a minimum of 1.6 g/kg of protein in the offseason, although targeting closer to 2.2 g/kg may ensure a more consistently optimized response across a greater proportion of athletes. 

Finally, among bodybuilders who struggle with offseason hunger and subsequently consume energy intakes that lead to faster rates of weight gain and excess fat accumulation, a higher protein intake may be useful (if not contraindicated for clinical reasons). In a study by Antonio and colleagues, resistance trained participants consuming more protein (4.4 g/kg per day) and more calories gained a similar amount of FFM, but did not gain additional body fat compared to a lower protein group consuming fewer calories [28]. Likewise, in a follow up study, a group consuming 3.4 g/kg of protein daily gained a similar amount of FFM, but lost a greater proportion of body fat compared to a lower protein group, once again, despite a higher energy intake [29]. The authors of these “free living” studies speculated their findings were due to increases in dietary induced thermogenesis via the very high protein diets. However, this is at odds with a more tightly controlled 2012 metabolic ward study by Bray and colleagues in which the protein content of the diet influenced the proportion of FFM gained, while total body mass was dictated by the diet’s energy content alone [30]. 

Thus, while dietary induced thermogenesis may indeed be meaningfully higher with protein intakes in the 3 g/kg or higher range, the fat loss or lack of weight gain observed by Antonio and colleagues, despite a reported higher energy intake, might also reflect the satiating effect of very high protein intakes decreasing actual energy intake, rather than an increase in thermogenesis alone.

### 3.2. Protein Quality

Essential amino acids (EAA) are the only amino acids required to stimulate the process of MPS [31]. While all amino acids provide the necessary “building blocks” for the synthesis of new tissue, the amino acid leucine in particular appears to be especially important as a “metabolic trigger” of MPS [32]. A sufficient concentration of leucine has been suggested to be necessary to reach a “leucine threshold” which is required to maximally stimulate MPS [33]. In short, from a muscle building perspective, protein sources that both trigger a robust MPS response (sufficient leucine quantity) and provide the essential building blocks for the construction of new muscle tissue (contain the full spectrum of essential amino acids in abundance) can be seen as “higher quality”. 

While the mechanistic effect of leucine on MPS is beyond the scope of this publication, readers are encouraged to read a review that covers this topic in detail [34]. In general, on a gram per gram basis, animal-based protein sources typically contain more leucine and EAA, although there are notable exceptions. Soy protein, one of the most common plant-based protein supplements, has all the EAA, but in a lower amount per gram compared to dairy protein and thus, in one study produced a smaller increase in MPS compared to whey after acute ingestion [35]. Interestingly, in this same study soy produced a larger increase in MPS than casein, also a “high quality” dairy protein, presumably due to the slower digestion speed of casein [35]. Meaning, while the leucine and EAA content of a protein source certainly should be considered, the acute MPS response is not the only variable linked to long term hypertrophy. Indeed, a high-quality but “slow” protein like casein produces a smaller amplitude MPS response initially. However, casein (and other slowly digested proteins) may produce a similar or larger MPS area under the curve when viewed longitudinally compared to a “fast” protein source like whey, which results in a larger initial increase and then a steep reduction [36]. 

More importantly, the acute MPS response to a given type of protein should not be viewed from a reductionist perspective. In the real world, multiple servings of various protein sources are consumed daily, likely making some of these distinctions in amino acid profile and digestion kinetics moot. Indeed, in a meta-analysis comparing longitudinal body composition changes with different types of protein supplements, there were no significant differences among participants consuming soy when compared to whey, other dairy proteins, or beef protein isolate [37]. 

As demonstrated in a study comparing groups consuming post-training protein (on top of a diet already consisting of 25% protein), whether 48 g of whey (containing 5.5 g of leucine) was provided, or 48 g of rice protein (containing 3.8 g of leucine) was provided, no impact was observed on body composition changes between groups after eight weeks [38]. Therefore, when consumed in sufficient quantities (especially considering total daily protein intake) the protein quality of an individual meal is of less concern. Even so, if one was to consume a diet dominated by plant-based protein sources, there are alternatives to soy and rice. For example, pea protein isolate is rich in both EAA and leucine. In a 12-week study, a group consuming 50 g of pea protein isolate daily had greater increases in resistance-training induced muscle thickness compared to placebo, which were not significantly different from a group consuming 50 g of whey [39]. 

Therefore, in the context of the recommendations in this article, protein quality may only be a concern if using the low-end range of the protein guidelines (1.6 g/kg), or if consuming a largely plant based diet. In either case, it might prove beneficial to supplement with leucine and EAA rich sources of protein—as appropriate based on dietary preference (e.g., dairy proteins or pea protein if vegan)—to ensure the expected MPS response to one’s protein intake occurs. 

## 4. Fats 

Fat is an essential nutrient vital for many functions in the body. However, less is known about the effect of dietary fat in regard to skeletal muscle hypertrophy. Intakes of dietary fat among bodybuilders have been reported to range from 8–33% of total calories [2]. Although intramuscular triglycerides can act as a fuel source during resistance training, they are not a limiting factor since substrates are derived primarily from anaerobic processes [40]. Of interest to the bodybuilder, there is evidence in endurance athletes [41] and hockey players [42] that low carbohydrate diets (30–45% of energy or lower) may affect the free testosterone to cortisol (fTC) ratio, which could have a negative impact on recovery. On the other hand, reducing dietary fat in isocaloric diets from ~30–40% to ~15–25% has resulted in significant but modest reductions in testosterone levels [43,44,45,46]. 

However, it is not clear that testosterone changes within normal ranges affect muscle gain significantly [47]. Despite the possibility that testosterone levels may be higher when consuming a greater proportion of energy from dietary fat, actual changes in muscle mass during longitudinal studies of resistance trained individuals following high fat, ‘ketogenic’ diets have consistently been inferior to moderate or lower fat approaches with ample carbohydrate [48,49,50,51]. Whether this is due to changes in exercise capacity, or alterations in fTC ratio, or some other mechanism related to the high fat or low carbohydrate component of the diet is yet to be elucidated. 

However, this indicates that perhaps a more moderate proportion of dietary fat should be consumed, rather than a low or high intake. In the literature, recommendations of 15–20% and 20–30% of calories from dietary fat have been proposed [15,52]. However, further research is needed to establish the effect and optimal amount of dietary fat for aiding muscle hypertrophy. 

Based on current evidence, it may be prudent to recommend that dietary fats should account for 20–35% of calories—conforming to The American College of Sports Medicine recommendations for athletes [53]—which under most circumstances would equate to approximately 0.5–1.5 g/kg/day. Further, it should be noted that sufficient intakes of dietary protein and carbohydrates should not be compromised by a high dietary fat intake. 

Fat quality such as omega 3 and omega 6 might also be of importance for bodybuilders. Provided sufficient intake from a high-quality diet containing good sources of these fatty acids, they do not need to be supplemented. However, it might be challenging for some to consume the optimal amounts. Thus, this will be discussed in further detail in the dietary supplements section.

## 5. Carbohydrates

Unlike proteins and fats, carbohydrates are considered non-essential for the human diet because the body has the ability to produce glucose needed by tissues through gluconeogenesis [54]. However, carbohydrate intake has an important role in the bodybuilder’s diet as a regulator of thyroid hormones and as a contributor to micronutrient needs [55,56]. Further, a very low carb diet could limit regeneration of adenosine triphosphate (ATP) and limit the muscles’ ability to contract with high force [57,58]. During high intensity exercise, muscle-glycogen is the major contributor of substrate and it has been shown that glycolysis provides ~80% of ATP demand from one set of elbow flexion when taken to muscular failure [59]. In spite of this, part of the glycogen used during this type of exercise can be resynthesized from lactate, which could reduce the carbohydrate requirement. Resistance training has also been shown to reduce muscle-glycogen by 24–40% in a single session [59,60]. 

The depleted amount may vary based on duration, intensity and the work completed, but typical bodybuilding training with higher repetition and moderate loads seems to cause the greatest reduction of muscle-glycogen stores [61]. Further, it has been suggested that when glycogen stores are too low (~70 mmol/kg), this may inhibit the release of calcium and hasten the onset of muscle fatigue [62]. Low muscle glycogen significantly reduces the number of repetitions performed when three sets of squats at 80% 1 RM are performed [57]. 

However, it has been shown that consuming a diet containing 7.7 g/kg/day of carbohydrate for 48 hours before a training session has no greater effect on performance compared to 0.37 g/kg/day when 15 sets of 15 RM lower-body exercise is performed [63]. Similarly, another study found that a 70% carbohydrate diet compared to 50% carbohydrate diet had no greater effect on performance during supramaximal exercise; however, a diet consisting of 25% carbohydrates significantly reduced performance [64]. 

Further, given the observed long term negative effects on muscle mass recently observed in trials of resistance-trained populations following ketogenic diets [49,51], it might be prudent for bodybuilders to simply ensure a sufficient intake of carbohydrates given these disparate results. Thus, while both moderate and high carbohydrate diets are likely appropriate for bodybuilding, very low carbohydrate diets may be detrimental to training. 

In male bodybuilders, average carbohydrate intakes of 5.3 g/kg/day have been reported during the off-season [2]. However, optimal amounts of carbohydrates have not been established for bodybuilders. In the literature, recommendations for strength sports, which includes bodybuilding, intakes of 4–7 g/kg/day and 5–6 g/kg have been proposed [15,65]. Carbohydrate seems to be important for the bodybuilder, but only moderate amounts may be required to yield benefits. Therefore, after calories have been devoted to protein (1.6–2.2 g/kg/day) and fats (0.5–1.5 g/kg/day), the remaining calories should be allotted to carbohydrates. However, based on current evidence, it might be reasonable to consume sufficient amounts of carbohydrates in the ≥3–5 g/kg/day range if possible. 

Further research is warranted among bodybuilders to conclude if habitually higher or lower carbohydrate intakes than have been observed might yield further benefits. Table 1 summarizes the recommendations for calories and macronutrients.

## 6. Nutrient Distribution and Timing

Bodybuilders are reported to have a mean intake of six meals a day [66]; however, there are no studies looking specifically at what might be an optimal meal frequency for this population [65]. This high frequency of meals is based on the belief of a greater state of anabolism and even a better use of nutrients during the day, which could translate into an improvement in body composition.

The concept of timing protein intake to maximize hypertrophy spans a number of dosing strategies. The first to appear in the literature was the consumption of protein in close proximity to resistance training. Peak MPS rates are higher in this period when protein is consumed; thus, this strategy is proposed to improve the efficiency of skeletal muscle repair and remodeling [31]. Additionally, due to the “muscle full effect”, whereby further provision of protein fails to increase MPS until sufficient time has passed, evenly spreading protein intake between multiple meals is another strategy designed to maximize total daily MPS [67]. Finally, pre-bed consumption of slow-digesting protein (such as casein) to prevent extended catabolic periods during sleep is the most recently proposed strategy to improve net daily protein balance [68]. Each of these three strategies will be discussed in turn. 

### 6.1. Protein Dosage

The post-training period permits a higher MPS peak when protein is consumed [31] and to reach peak MPS, an adequate “threshold” leucine dose may be needed [32]. Several studies have examined the protein dosage required to maximize MPS after training [69,70,71]. In one, 0, 5, 10, 20 or 40 g of whole egg protein was consumed following lower-body resistance exercise with 20 g maximally stimulating MPS [69]. Similar results were also seen in another study, where 20 g whey was sufficient to maximally stimulate post-absorptive rates of MPS both at rest and after unilateral leg work at 80% of 1 RM [70]. Further, 40 g of whey produced no additional increases of MPS in this study and lead to oxidation and urea production. 

However, a recent study found that when performing whole-body resistance exercise at 75% of 1 RM, 40 g of whey produced a significantly higher MPS response compared to 20 g [71]. Therefore, there is a relationship between the volume of muscle tissue that is damaged and stimulated, and the appropriate intake of protein. Interestingly, authors of a 2013 meta-analysis noted that despite short term tracer studies showing greater MPS responses when protein was consumed in the “window of opportunity” post-training, in longitudinal training studies no significant effect on hypertrophy was found when controlling for total daily protein intake regardless of whether protein was consumed within the window, or outside it [72]. 

### 6.2. Nutrient Timing

Similarly, researchers in a short term tracer study investigating protein dosing over the course of 12 hours reported a greater MPS area under the curve when four 20 g whey protein doses were consumed every three hours compared to two 40 g doses six hours apart and eight 10 g doses every hour and a half [73]. In theory, given the threshold past which additional protein consumed in a single sitting does not further contribute to MPS [69], and due to the post-prandial “refractory period” during which MPS cannot be maximally stimulated again [67], one would conclude that a bodybuilder should reach—but not exceed—this threshold dose every few hours to maximize long term hypertrophy. However, authors of a 2018 systematic review on protein supplements including 34 randomized controlled trials, reported similar lean mass gains among groups using a with-meal (resulting in fewer protein servings of a high magnitude) and between-meal (resulting in more protein servings of a moderate magnitude) dosing schedule [74]. 

Intriguingly, data examining night-time protein feedings display a similar disconnect between short term mechanistic studies and long-term training interventions. In 2012, the first research examining the acute response to night-time casein feeding was carried out [68]. In it, the authors reported 40 g of casein consumed before bed was digested, absorbed, and stimulated MPS and improved whole-body protein balance during the overnight period to a greater degree than placebo. Additional acute studies were published in the years following which confirmed [75] and also reconfirmed these findings in an older population [76]. In 2015, authors of the first longitudinal study reported enhanced strength and hypertrophy in a night-time protein-supplemented group compared to a placebo group [77]. 

However, total daily protein was not matched, as the night-time protein group consumed 1.9 g/kg/day while the placebo group only consumed 1.3 g/kg. Importantly, in both of the only protein matched longitudinal studies comparing night-time casein supplementation to earlier-supplemented groups, no significant differences in FFM gains were reported between groups [78,79]. Thus, the question is the same for each distribution strategy, why are there repeated disconnects between short term mechanistic studies of MPS and long-term research examining actual hypertrophy? The answer may lie in the methods used in MPS studies as participants are fasted, provided only protein powder in isolation, often given whey (which is digested very quickly) and observed for short periods. These lab settings result in different digestion time courses and amino acid kinetics than occur in the “real world”. Specifically, in these lab conditions baseline levels of amino acids in the body are lower than normal, and digestion and subsequent delivery of amino acids to muscle is faster. 

In free-living conditions, protein is consumed primarily from whole food sources, multiple times per day, and in conjunction with other foods, all of which delays gastric emptying. For these reasons, amino acids are titrated into the bloodstream in a slower, more consistent manner; thus, there is almost always a readily available supply under normal conditions [80]. Therefore, the effectiveness of the “anabolic window” and even protein distribution strategies might not translate to practice. Additionally, lab-specific limitations extend to night-time feeding studies as well. Consider for example, that 26 g of protein from lean steak results in a sustained elevation in MPS lasting at least six hours (the entire time period studied) [81]. 

Furthermore, 26 g is only ~37% the protein dose contained on average in an American dinner [82], which would take longer to digest due to the larger serving of protein, and the addition of fiber, lipids and other nutrients which would further delay digestion [80]. Therefore, the typical final meal may already fulfil the intended purpose of a casein shake. With that said, despite these disconnects between MPS and body composition outcomes, there is certainly no harm from attempting these strategies, especially if implemented in a pragmatic manner that doesn’t introduce additional logistical strain on one’s daily schedule. 

Therefore, it might be prudent to recommend bodybuilders to divide their daily intake of 1.6–2.2 g/kg of protein per day into multiple meals each containing ~0.40–0.55 g/kg [80] and ensure that one of these meals occurs within 1–2 hours before or after training, and one feeding consisting of a non-whey protein source is consumed 1–2 hours prior to sleep. For example, a 90 kg bodybuilder might consume 40–50 g of protein at 8–9 am for breakfast, train at 11 am, have 40–50 g of protein at 12–1 pm for lunch/post-training, 40–50 g of protein at dinner between 5–6 pm, and then a final meal of 40–50 g of non-whey protein at 9–10 pm before heading to bed by 11 pm. 

Carbohydrates consumed peri-workout is often a strategy utilized by athletes to improve performance in high intensity exercises. Complete glycogen resynthesis can be achieved within 24 hours following a glycogen depleting training bout if sufficient amounts of carbohydrate are consumed [83]. However, only 24–40% of muscle glycogen is depleted following resistance exercise [59,60]. Therefore, an amount of ≥3–5 g/kg carbohydrates per day would most likely be enough for glycogen resynthesis. This high daily carbohydrate intake likely also reduces the impact of pre-workout carbohydrate timing on exercise performance. 

Consuming carbohydrates with protein post-workout is often claimed to have a an anabolic effect due to the secretion of insulin. Although insulin has been shown to have anabolic effects [84], at physiological levels its release has little impact on post-exercise anabolism [85]. Further, several studies have shown no further effects on muscle protein synthesis post-exercise when carbohydrates are combined with amino acids [86,87]. 

In addition to bodybuilders lacking the need to emphasize glycogen replenishment, protein enhances post workout MPS to maximal levels even without the addition of carbohydrate [86,87]. While there is certainly no harm in post-workout carbohydrate consumption, doing so is unlikely to enhance long term hypertrophy as discussed in prior reviews [1,88]. Therefore, it may be best to focus on consumption of adequate daily carbohydrate and base carbohydrate distribution around the workout on personal preference.

## 7. Dietary Supplements 

In a recent survey among bodybuilders, it was reported that all of the participants were taking dietary supplements [9]. The most common dietary supplements were: protein supplements (86%), creatine (68%), branched chain amino acids (67%), glutamine (42%), vitamins (40%), fish oil (37%) and caffeine/ephedrine containing products (24%). 

Although protein supplements are popular among bodybuilders, they are predominantly used in the same way as whole foods to reach protein targets. Therefore, they will not be discussed in further detail. Readers are encouraged to read the ISSN position stance on this topic [89]. Further, covering all supplements commonly used by bodybuilders is beyond the scope of this review. Rather, the focus will be on dietary supplements that might potentially yield an ergogenic effect and supplements that can insure sufficient intake of micronutrients and essential fatty acids. 

### 7.1. Creatine Monohydrate 

Creatine phosphate is found in high concentrations in skeletal and cardiac muscle where it acts as an energy source [90]. Creatine can also be obtained through the diet in individuals who consume meat; however, creatine concentrations in meat are reduced with cooking [91]. 

Numerous studies have observed increases in muscle mass and strength following creatine loading phases typically of 20 g daily for around 1 week oftentimes followed by maintenance phases of 2–3 g creatine daily [92]. However, the loading phase may not be necessary. Muscle creatine saturation following 3 g creatine monohydrate supplementation for 28 days was shown to be similar to creatine monohydrate consumption following the typical loading phase [93]. 

Most individuals do not reach 3 g daily through the diet and supplementation may be necessary. There are numerous forms of creatine in supplements on the market of which creatine monohydrate is the most studied. Newer versions of creatine such as kre-alkalyn [94] and creatine ethyl-ester [95] have not been shown to be superior to creatine monohydrate despite typically having a higher price point. Therefore, we recommend consumption of 3 g creatine monohydrate daily. Timing of creatine does not seem to matter as saturation of creatine phosphate stores takes approximately 28 days to reach maximum concentrations when 3 g/day is consumed and does not have an acute effect [93].

### 7.2. Caffeine

One of the most used dietary supplements among bodybuilders are stimulants, in particular caffeine [9]. In addition to increasing arousal [96], caffeine can reduce pain and perceived exertion during exercise [97] and improves calcium handling which may increase power output [98]. Studies on resistance exercise have found that caffeine reduces fatigue and increases strength [99,100]. However, not all studies have shown an ergogenic effect on resistance exercise [101]. Studies that have shown an ergogenic effect have used high dosages of caffeine (5–6 mg/kg) which is at the upper limit of what is considered a safe dosage [99,100]. However, it may be advisable to consume the minimum effective dosage for an individual as tolerance can arise from regular intake [102]. Due to the acute effect of caffeine, it is advisable to consume caffeine approximately 1 hour before exercise [99]. However, the half-life of caffeine is roughly 3–9 hours; therefore, it may be advisable to consume caffeine earlier in the day to support healthy sleep patterns if exercise is performed later in the day [103]. Further research is warranted for a consensus on the use of caffeine regarding resistance exercise but based upon the current evidence a dosage of 5–6 mg/kg consumed pre-exercise might yield an ergogenic effect on resistance exercise performance. 

### 7.3. Beta-Alanine

Ingestion of 4–6 g beta-alanine has been shown to elevate muscle carnosine levels [104]. Carnosine acts as a pH buffer in skeletal muscle and may delay the onset of muscle fatigue during high-intensity exercise [105]. A meta-analysis concluded that beta-alanine might yield ergogenic effects during high-intensity exercise lasting 60–240 seconds [104]. Further, there were no beneficial effects in exercise lasting <60 seconds. Most of the studies included in the meta-analysis looked at endurance exercise. 

However, there is evidence that beta-alanine supplementation may improve muscular endurance in resistance-trained athletes [105] and may improve body composition [106]. Further studies are warranted to examine the ergogenic effect of beta-alanine on body composition and performance. However, given that bodybuilders often train with more than 10 repetitions per set and often times include intensity techniques such as drop sets, rest pauses, myo reps and others, beta alanine might yield a benefit in the endurance of these sets [9]. 

Thus, it might be reasonable for a bodybuilder to consume 3–5 g beta alanine daily during high repetition training phases or training phases where they are incorporating several intensity techniques that prolong the duration of a set. Similar to creatine monohydrate, beta-alanine does not have an acute effect as muscle carnosine concentrations takes approximately 4 weeks to reach concentrations that would yield an ergogenic effect, provided that sufficient amounts are consumed daily [104].

### 7.4. Citrulline Malate

Recently, citrulline malate has gained popularity among bodybuilders. The potential ergogenic effect is thought to be increased ATP production and citrulline malate’s potential ability to act as a buffering agent [107]. Consumption of 8 g citrulline malate has been shown to increase repetitions to failure by as much as 50 percent [107,108,109,110], decrease muscle soreness by 40 percent [107] and improve maximal strength and anaerobic power [111]. 

However, not all studies have observed ergogenic effects of citrulline malate consumption. Two recent studies failed to show improvement in performance, augment the muscle swelling response to training, alleviate fatigue or increase focus and energy following citrulline malate supplement in recreational resistance trained men [112,113]. 

A recent meta-analysis by Trexler et al. analyzed 12 studies on CM for strength and power performance [114]. Although they only found a small effect size (0.20), they concluded that this might be relevant for high level athletes where competition outcomes are decided on small margins, such as high level competitive bodybuilders. It is advised to consume citrulline malate approximately 60 min before exercise to allow for sufficient absorption.

Further research is warranted to determine the efficacy of citrulline malate for resistance exercise. At this stage, the data indicates either a beneficial or neutral effect on performance. Thus, based on current evidence, 8 g/day of citrulline malate consumed pre-exercise might have some benefits that are of interest to bodybuilders. 

### 7.5. Multivitamin/Mineral

Historically, bodybuilders have utilized restrictive diets that eliminate foods or entire food groups. As a result, numerous vitamin and mineral deficiencies are common. In dieting bodybuilders, deficiencies including calcium, vitamin D, zinc, iron and others have been observed [115,116,117]. However, a majority of literature on dietary practices of bodybuilders is from the 1980’s and 1990’s; therefore, more recent data is needed [2].

More recently, dieting practices in bodybuilders who use a traditional restrictive diet were compared to competitors using a macronutrient-based dieting approach where no food or food group was off limits [118]. Not surprisingly, competitors using a more flexible dieting approach were found to have fewer micronutrient deficiencies. Specifically, vitamin E, vitamin K, and protein were found to be significantly lower in women utilizing strict dietary approaches compared to those using more flexible approaches. In the current review, we recommend using a flexible dieting approach where no food or group is eliminated from the diet. 

Thereby, micronutrient deficiencies are less likely to occur, especially considering that competitors in the offseason have a greater caloric allotment than those dieting for a show which should allow them to incorporate a greater variety of foods. 

Nevertheless, it may be advisable to recommend a low dose multivitamin/mineral supplement (≤100% RDA) as a failsafe to prevent any major micronutrient deficiencies while also emphasizing consumption of a variety of foods daily to meet micronutrient needs.

### 7.6. Omega 3

Polyunsaturated fatty acids with a double bond three atoms away from their terminal methyl group are known as ω-3 or omega-3 fatty acids (O3). Low intakes of O3 in western diets in relation to other sources of dietary fat (such as omega-6 fatty acids) are associated with poorer multi-spectrum health in epidemiological studies [119]. Thus, specific focus on dietary changes to supply eicosapentaenoic and docosahexaenoic acids (EPA and DHA)—the dietary shortfall most common in the western world—is of interest; but it is worth noting the measurement, interaction, and effect of O3 and omega-6 fatty acids in relation to health is unclear and beyond the scope of this article. Readers are referred to [120] for a review.

In addition to health, there is interest regarding the potential anabolic effects of EPA and DHA supplements [121] which are typically supplied via fish oil or in some cases algae oil. However, there are mixed data on fish oil’s ability to augment the muscle protein synthesis response to protein ingestion. While a 2014 review paper highlighted a number of studies which found fish oil can enhance the response [122], a recent study found no effect on the MPS response to a resistance training session and post-workout protein ingestion [123]. More importantly, data on longitudinal hypertrophy are few [124] and studies on resistance training performance are mixed [125] and largely not applicable or difficult to appraise due to the use of untrained participants or non-standardized, ecologically unrealistic training relative to bodybuilding.

In a recent review specifically addressing the question of whether or not O3 supplements might enhance hypertrophy [126], the authors concluded there is not currently sufficient evidence to make such a claim. While additional research is needed before O3 supplementation (or diet alterations for that matter) can be recommended for muscle-building purposes, the health benefits of O3 supplementation are worth noting. For example, recent meta-analyses have reported fish oil supplementation reduces symptoms of depression [127], decreases risk of cardiac death [128], decreases blood pressure [129], and decreases waist circumference [130]. Therefore, physique athletes may consider fish (or algae) oil supplementation daily (2–3 g EPA/DHA) for general, multi spectrum health, but future study is needed to make recommendations regarding bodybuilding performance. Table 2 summarizes recommendation for dietary supplements.

## 8. Summary

Bodybuilders in the off-season should focus on consuming a slightly hyper-energetic diet (~10–20% above maintenance calories) with the aim of gaining ~0.25–0.5% of bodyweight per week. Advanced bodybuilders are advised to be more conservative with the caloric surplus and the rate of weekly weight gain. Dietary protein intake is recommended to be 1.6–2.2 g/kg/day with a focus on sufficient protein at each meal (0.40–0.55 g/kg/meal) and an even distribution throughout the day (3–6 meals). Dietary fats should be consumed at moderate levels, neither too low nor high (0.5–1.5 g/kg/day), to prevent an unfavorable fTC ratio and to prevent reductions in testosterone levels. After calories has been devoted to protein and fat, the remaining calories should come from carbohydrates while ensuring sufficient amounts are consumed (≥3–5 g/kg/day). Minor benefits can be gained by consuming protein (0.40–0.55 g/kg/meal) in close proximity to training sessions (1–2 hours pre-exercise and within 1–2 hours post-exercise). CM (3–5 g/day), and caffeine (5–6 mg/kg) should be considered as they can yield ergogenic effects for bodybuilders. Further, BA (3–5 g/day) and CITM (8 g/day) are dietary supplements that can be considered as they may potentially be of benefit for bodybuilders, depending on individual training regimens. Bodybuilders who are unable to consume a sufficient intake of micronutrients and essential fatty acids in their diets should consider supplementing these nutrients to avoid deficiencies. The primary limitation of this review is the lack of large-scale and long-term studies on bodybuilders in the off-season. Further research is warranted in this population to optimize nutrition and dietary supplement recommendations. 

## Figures and Tables

**Table 1:** Dietary recommendation for bodybuilders in the off-season.

Diet Component	Recommendation Novice/Intermediate	Recommendation Advanced
Weekly weight gain	~0.25–0.5 (% of body weight)	~0.25 (% of body weight)
Calories	+10–20% above maintenance	+5–10% above maintenance
Protein	1.6–2.2 g/kg	1.6–2.2 g/kg
Fats	0.5–1.5 g/kg	0.5–1.5 g/kg
Carbohydrates	Remaining calories (≥3–5 g/kg)	Remaining calories (≥3–5 g/kg)

**Table 2:** Recommendations for dietary supplements and dosage for bodybuilders.

Dietary Supplement	Recommended Dosage
Creatine monohydrate	3 g/day
Beta-alanine	3–5 g/day
Citrulline malate	8 g/day
Caffeine	5–6 mg/kg
Multivitamin/mineral	Low dose micronutrient supplement (≤100% RDA)
Omega 3	2–3 g EPA/DHA
